# Automated Pneumonia Based Lung Diseases Classification with Robust Technique Based on a Customized Deep Learning Approach

**DOI:** 10.3390/diagnostics13020260

**Published:** 2023-01-10

**Authors:** Yaman Akbulut

**Affiliations:** Department of Software Engineering, Faculty of Technology, Firat University, Elazig 23200, Turkey; yamanakbulut@firat.edu.tr; Tel.: +90-424-607-4282

**Keywords:** ILD, MCW segmentation, customized deep learning

## Abstract

Many people have been affected by infectious lung diseases (ILD). With the outbreak of the COVID-19 disease in the last few years, many people have waited for weeks to recover in the intensive care wards of hospitals. Therefore, early diagnosis of ILD is of great importance to reduce the occupancy rates of health institutions and the treatment time of patients. Many artificial intelligence-based studies have been carried out in detecting and classifying diseases from medical images using imaging applications. The most important goal of these studies was to increase classification performance and model reliability. In this approach, a powerful algorithm based on a new customized deep learning model (ACL model), which trained synchronously with the attention and LSTM model with CNN models, was proposed to classify healthy, COVID-19 and Pneumonia. The important stains and traces in the chest X-ray (CX-R) image were emphasized with the marker-controlled watershed (MCW) segmentation algorithm. The ACL model was trained for different training-test ratios (90–10%, 80–20%, and 70–30%). For 90–10%, 80–20%, and 70–30% training-test ratios, accuracy scores were 100%, 96%, and 96%, respectively. The best performance results were obtained compared to the existing methods. In addition, the contribution of the strategies utilized in the proposed model to classification performance was analyzed in detail. Deep learning-based applications can be used as a useful decision support tool for physicians in the early diagnosis of ILD diseases. However, for the reliability of these applications, it is necessary to undertake verification with many datasets.

## 1. Introduction

Around the world, acute infections of the lower respiratory tract have been a major source of illness and death [1]. Millions of people each year are impacted by lung disease, which poses serious hazards to children, seniors 65 and over, and those with a variety of clinical cases containing obesity, diabetes, and high blood pressure. Different factors can bring about lung disease, and the most known cause is viral [2].

A new member of the infectious lung disease back (ILD), COVID-19, first appeared in Wuhan, China at the end of 2019. The ICTV (International Committee on Taxonomy of Viruses) initially determined the coronavirus as SARS-CoV-2 [3]. At the beginning of 2020, the WHO (World Health Organization) changed its name to COVID-19. In March 2020, COVID-19 was named by the WHO as a pandemic disease. The number of COVID-19 diseases and fatalities surged so quickly during the pandemic that they reached approximately 600 million and 6.5 million, respectively [4]. The novel coronavirus has spread throughout the world due to this rise in instances.

Different signs and symptoms of infection, including high fever, diarrhea, coughing, respiratory conditions, and weariness, can be caused by the COVID-19 disease. In some active cases, COVID-19 can result in the patient experiencing major issues such as breathing difficulties, multi-organ failure, pneumonia, abrupt cardiac arrest, and even death. Because of the exponential boost in the number of active cases, healthcare services had virtually disappeared in many affluent nations. Until COVID-19 vaccines were created, most nations lacked testing supplies and adequate ventilators. The COVID-19 virus also made the situation more urgent. Many nations have cut off access to other nations because of this. These nations also pushed their citizens to stay at home and discouraged them from traveling domestically or internationally [5]. Despite the COVID-19 vaccines appearing to have brought the pandemic under control, the disease is still prevalent because fewer individuals are choosing to wear masks and more people feel comfortable going out in public. It is also of great importance that pneumonia, one of the most established ILD diseases, can be accurately distinguished from the popular COVID-19 disease.

Isolating infected patients from those who are not sick is one of the most crucial strategies in the fight against ILD. The most reliable and practical approach to diagnosis is chest X-ray (CX-R), which is a radiological imaging technique [6,7].

In recent times, the ILD with COVID-19 disease is a hot topic among scientists from many different academic fields around the world. Some researchers have submitted publications describing artificial intelligence-based algorithms for automatic ILD categorization from computed tomography (CT) and CX-R images to assist radiologists and specialists in making decisions [8,9,10].

In this study, it was aimed to improve the classification performance for ILD, particularly COVID-19, as it has severely impacted the human health system. Therefore, a specific deep-learning technique was developed for automated classification. The contributions of the proposed approach were expressed as follows:

- Different regions on images are marked using the MCW segmentation algorithm. Because of this, it enables the unique information in the data to stand out. The pre-processing operation with the MCW algorithm increased the classification accuracy.
- The attention structure in the CNN models is used to increase the distinctive representation. The LSTM blocks in deep learning models are added to benefit the ability to keep weight information in their memory blocks. Therefore, the attention-CNN LSTM (ACL) model, which was synchronously trained in the attention structure, convolutional layers, and the LSTM model, improved classification performance compared to the CNN model which did not contain attention and LSTM structures.

## 2. Related Works

Particularly in the medical field, numerous computer-aided detection methods have advanced substantially during the past few decades. Several artificial intelligence (AI)-based deep learning algorithms have been used in numerous medical applications, most notably in detection and diagnosis. Recent years have seen success with AI in the identification of several illnesses, including plant disease [11], osteoporosis [12], breast cancer [13], cardiovascular disease [14], and poultry disease [15]. Systems for computer-aided, deep learning-based ILD identification containing COVID-19 disease are necessary since ILD is now a popular clinical problem. Therefore, numerous researchers have created different AI applications employing both X-ray and CT images. Given that X-ray exams are less expensive than CT scan exams, it is practical and cost-effective to identify ILD utilizing CX-R images. On an X-ray dataset, Afshar et al. developed the COVIDCAPS framework, which has a 95.7% accuracy, and a 95.8% specificity [16]. These applications are capable of handling even little datasets with efficiency. Similar to how ResNet50 and Inception versions were utilized to build other models, the highest 99.7% accuracy was obtained by the ResNet50 model for binary classification [8]. Sethy et al. [17] successfully obtained an accuracy of 95.38% when separating the COVID-19-positive patients from the other cases using the SVM with the ResNet50 using learnable features from X-ray images.

Additionally, a deep convolutional neural network design has been applied to CX-R images by several researchers, producing accurate and useful results [9]. Hemdan et al. [18] built a customized CNN model for automated ILD classification. The structure containing seven CNN made up the proposed model. For binary and multi-class (pneumonia, COVID-19, and healthy) categorization, Apostolopoulos et al. [19] attained an accuracy of 98.75% and 93.48%, respectively. To classify the ILD samples including 1427 X-ray images, their deep learning model applied transfer learning. Utilizing data from multimodal imaging, Horry et al. [20] conducted detection through transfer learning. With the right parameters, the selected VGG19-based transfer learning model was able to achieve an 86% precision for ILD from ultrasounds (multi-class classification) and an 84% precision for CT images (binary classification). Using the DarkCovidNet network, Ozturk et al. [21] achieved an accuracy of 98.08% and 87.02% for ILD databases consisting of binary- and multi-classes. There are 17 CNN layers in all, each with a unique set of filters. Learning parameters were updated by the chaotic squirrel search algorithm, and the prediction process was carried out using the EfficientNet-B0 network, another hybrid model created by Altan and Karasu [22]. Transfer learning has been employed by Tsiknakis et al. [23] to categorize COVID-19 and standard X-ray images. They have determined that the entire Receiver Operating Characteristics (ROC) curve area is equal to 1. Demir [24] presented a hybrid deep learning model, which combined convolutional layers and the LSTM model, to automatically classify ILD. The model, named the DeepCoroNet, reached a classification accuracy of 96.54%. Ismael and Sengur [25] used a ResNet50 based-transfer learning approach for binary ILD classification. Deep features were extracted from the ResNet50 model. COVID-19 samples with deep features conveyed to the SVM algorithm were classified with an overall 94.7% accuracy. Muralidharan et al. [26] utilized a new deep-learning approach for automated ILD detection from X-ray images. First, X-ray image levels containing seven modes were tuned with a wavelet transform-based algorithm. To classify healthy, COVID-19, and pneumonia samples, these multiscale images were transmitted to the multiscale deep CNN. An accuracy of 96% was obtained with this model. Demir et al. [27] proposed a deep autoencoder that consisted of convolutional layers and an autoencoder model for ILD classification. The compressed layer (pooling layer) representation of the deep autoencoder network was used to extract features. A multilevel feature selection algorithm named serial data analysis and regression (SDAR) reduced the feature set sizes and boosted classification achievement. The classification accuracy of 97.33% was performed by the SVM classifier.

A good classification performance could not be obtained with CNN-based approaches trained from scratch when the approaches related to ILD are examined in general. A classifier such as SVM is also used to improve classification performance. This has increased the computational cost in the classification process. In the proposed study, superior performance has been achieved without the need for a separate classifier by increasing the performance of CNN-based models with attention and residual structures.

## 3. Dataset

**Figure 1:**
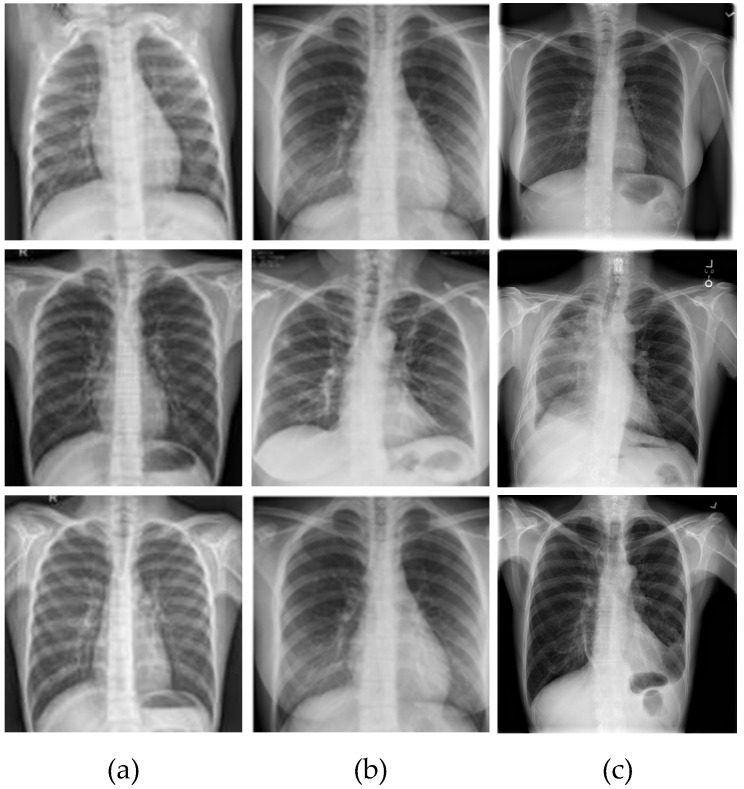
The samples of the dataset for each class: (**a**) Normal, (**b**) COVID-19, and (**c**) Pneumonia.

The ILD database that was used contained 1061 CX-R samples in total, gathered from various accessible public sources. Radiologists and other specialists carried out the labeling activities. The COVID-19, Normal, and Pneumonia folders were used to reorganize the CX-R images. The numbers of COVID-19, Normal, and Pneumonia samples were 361, 200, and 500, respectively. Of the COVID-19 cases, 161 were female, compared to 200 male cases, and the average age of the individuals was above 45. The combined database with the COVID-19 and typical (healthy) CX-R samples were collected from the Kaggle database website links [28,29]. The dataset created by Wang et al. [30] added samples from the pneumonia class. Figure 1 displays CX-R image samples for each class. In Figure 1, the normal, COVID-19, and pneumonia classes are represented by the CX-R image samples included in the first, second and third columns respectively.

## 4. Proposed Methodology

**Figure 2:**
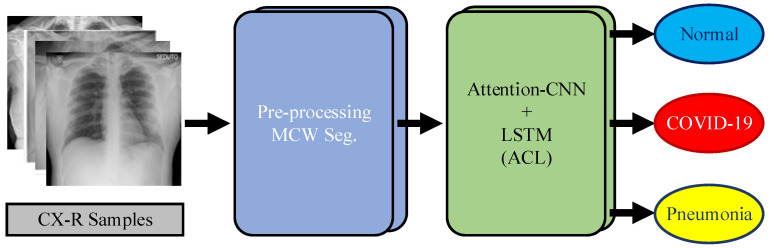
The framework of the proposed approach.

In this study, a novel and efficient method for highly accurate ILD detection was developed. The dataset consisting of CX-R samples was used to evaluate the suggested approach, shown in Figure 2. Processing with marker-controlled watershed (MCW) segmentation of CX-R samples, and the attention-CNN LSTM (ACL) model, were the two steps of the suggested methodology. The CX-R images were subjected to pre-processing procedures at the initial level to improve classification performance. Gradient operation employing the Sobel operator was the initial level in the pre-processing procedure. The CX-R samples’ blob regions were highlighted using the gradient operator. In other words, the performance of the MCW segmentation was enhanced by the application of the gradient operator. The blobs on the gradient images were segmented using the MCW segmentation at the following level. Segmentation was utilized to lessen gray regions in the CX-R sample. In the third level of the pre-processing, CX-R samples were resized to 100 (height) × 100 (width) for standardizing CX-R samples and reducing the computational cost. In the last step, the processed CX-R samples were transmitted to the ACL model, which consisted of the attention structure, convolutional layers, and the LSTM model. The attention structure in the ACL model was used to increase the distinctive representation of the highlighted CX-R samples using the MCW segmentation algorithm. The convolutional layers were utilized to extract significant feature maps of CX-R samples. The LSTM blocks in the ACL architecture were added to benefit the ability to keep weight information in their memory blocks. These three strategies in the ACL model were synchronously operated in the training stage.

## 5. Methodology Techniques

### 5.1. Pre-Processing

The directional gradient is used to compute the gradient magnitudes and directions for input images in the gradient method. When performing these gradient operations, a gradient operator like Sobel, Roberts, and Prewitt [31] is used. Surface pixel density, including light pixel density, is high in the watershed transform. In other words, surfaces with low pixel density include dark surfaces. The watershed transformation can be used to identify catchment basins (CatBas) and watershed ridge lines in a sample [32]. The catchment basin $CatBas(m_{j})$ Equation (1) of a minima $m_{j}$ is defined in the context of the watershed transformation as the collection of values (x) that are topographically nearest to $m_{j}$ compared to other local minimum $m_{i}$ in watershed transformation where function f∈CatBas(D) has minimum ${\left\{m_{k}\right\}}_{k\in S}$ for a set S:

(1) $$CatBas\left(m_{j}\right)=\{x\in D\;\;|\nabla i\in S\left\{i\right\}:\;\;\;f\left(m_{j}\right)+T_{d}\left(x,m_{j}\right)<f\left(m_{i}\right)+T_{d}\left(x,m_{i}\right)\}$$

where domain and topographical distance, respectively, are D and $T_{d}$. The set of points with no relation to any CatBas is known as the watershed transformation of $f\left(W_{shed}\left(f\right)\right)$ Equation (2):

(2) $$W_{shed}\left(f\right)=D\cap \left(\cup_{j\in S}CatBas\left(m_{j}\right)\right)$$

given that $W_{shed}$ is a tag, $W_{shed}\notin S$, and $W_{shed}\left(f\right)$ is a mapping, and $\beta :D\to S\cup W_{shed}$ is the result.

A strong and reliable algorithm for separating items with covered shapes, those whose borders are described as ledges, has been identified as the MCW segmentation. The associated objects have markers added to them. The associated items and backgrounds are given the inner and outer markers, respectively. By separating each object from its neighbors after segmentation, watershed zones are created on the selected ledges. As a result, the MCW segmentation algorithm can distinguish each distinctive tiny or large detail in a radiological image at the regional level. The MCW segmentation technique contains the following steps:

Step-1 Calculate the segmentation process that divides dark areas into items.
Step-2 Determine the foreground markers, which contain the linked pixel blots inside of each object.
Step-3 Determine background markers or pixels that are not a part of any item.
Step-4 Update for decreasing the foreground and background marker locations’ segmentation functions.
Step-5 Use the revised parameters to calculate the watershed transform.
Step-6 Compute learning parameters.

### 5.2. Machine Learning Technique

In the sequence folding layer, a set of image queue data is converted into a group of images, and convolution procedures are then implemented to these image queue data by employing a period. The data from the sequence folding layer is turned into sequence structure in the sequence unfolding layer.

The fundamental structural layer for a CNN called the convolution layer uses the convolution operation [33]. In this layer, there are several learnable filters. Convolutional layers extract features from inputs that are present in local, related parts of the dataset and assign their perspective to a feature map.

The implementation of the batch normalization (BN) layer is done to speed up network initialization and cut down on training time. Additionally, the vanishing gradient problem is lessened by employing BN layer operations [34]. The ReLU layer serves as the activation function and is used to set the gradient vanishing and explosion problems [35].

2-D data from the convolutional structure is converted into 1-D data through the smoothing layer to be used in the LSTM structure [36]. Classical LSTM layers consist of controlled structure units with input, output, and forget gates [37]. LSTM layers hold information data that were decided upon in a prior period and regulated the data transfer in units by using these gates. LSTM layers also significantly reduce the gradient disappearing and explosion issues. The forget gate structure resembles a neural network containing single-layer. Equation (3) states that the forget gate is active when the output is one.

(3) $$f_{t}=\sigma \left(W\left[x_{t},h_{t-1},C_{t-1}\right]+b_{f}\right)$$

where the logistic sigmoid function is σ, the weighted vector is W, and the biased values are $b_{f}$, the output vector of the preceding LSTM unit is $h_{t-1}$, the prior LSTM unit memory is $C_{t-1}$, and the accessible LSTM unit input is $x_{t}$.

The existing memory in the input gate’s structure is made up of a single-layer neural network with the values of the previous memory units and the hyperbolic tangent function. Equations (4) and (5) present the respective formulae.

(4) $$i_{t}=\sigma \left(W\left[x_{t},h_{t-1},C_{t-1}\right]+b_{i}\right)$$

(5) $$C_{t}=f_{t}\cdot C_{t-1}+i_{t}\cdot \tan h\left(\left[x_{t},h_{t-1},C_{t-1}\right]\right)+b_{c}$$

The output gate receives the transmission of data and information from the current LSTM layer. Equations (6) and (7) show the computations for the output gate.

(6) $$\sigma_{t}=\sigma \left(W\left[x_{t},h_{t-1},C_{t-1}\right]+b_{o}\right)$$

(7) $$h_{t}=o_{t}\cdot \tan h(C_{t})$$

The fully connected (FC) layer connects all of the neurons that are in the upper and lower layers. Neuron values are used to determine compatibility information for value and class [38]. The softmax layer receives the final FC layer data, including class possibility outcomes. The drop-out layer prevents the over-fitting issue by equating a set of input values to zero with a specified probability during optimization operation in training [39]. The softmax function Equation (8) for classifying in CNNs, performs the following functions:

(8) $$S^{k}=\frac{e^{x_{k}}}{\sum_{i=1}^{N}e^{x_{i}}}$$

**Figure 3:**
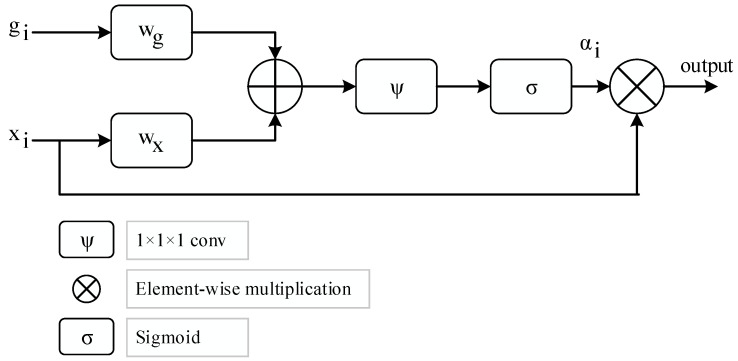
The framework of attention strategy.

The attention structure utilized in the proposed model is given in Figure 3, where $g_{i}$ depicts a gating signal vector acquired at a coarser scale and $x_{i}$ represents the output feature map of the *i*th layer, which subsequently sets the focus region for each pixel [40]. Equations (9) and (10) provide the computation of out using element-wise multiplication.

(9) $$out=\alpha_{i}\times x_{i}$$

(10) $$\alpha_{i}=\sigma \left(\varphi^{T}\left(w_{x}{}^{T}x_{i}+w_{g}{}^{T}g_{i}+b_{g}\right)+b_{\varphi }\right)$$

Bias terms are $b_{\varphi }$ and $b_{g}$ where linear transformations are w and φ using the 1 × 1 × 1 dimensional convolution operator, respectively. The learnable parameters for the attention modules are initially set at random and are optimized from scratch.

## 6. Experimental Studies

**Figure 4:**
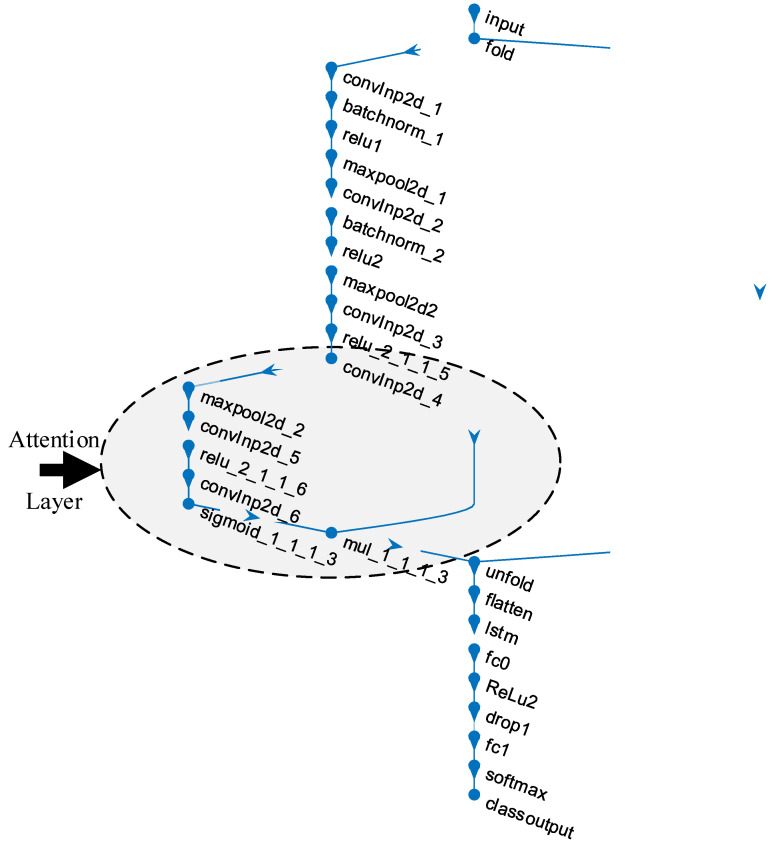
The representation of the proposed ACL model.

Coding procedures were operated on the Matlab R2021a program installed in a Windows-based operating system (Win 10 Pro) equipped with an Intel Core i9 processor, 32 GB DDR5 RAM, and 4 GB graphics card. Figure 4 shows the layer representation of the ACL network. The convolutional structure (six convolutional layers) in the ACL model starts with the convolutional layer named convlnp2d_1 and ends with the convolutional layer named convlnp2d_6. The attention structure in the ACL model was designed from the convlnp2d_4 convolutional layer.

The detailed layer information of the 28-layer ACL model is given in Table 1 in a sequential layer architecture.

**Figure 5:**
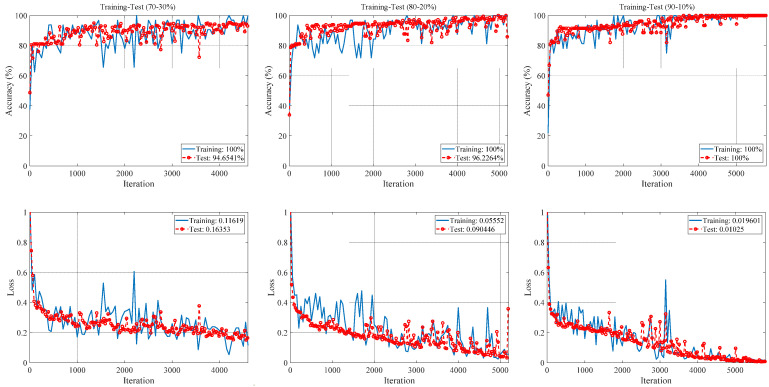
The accuracy and loss graphs for different training-test ratios of the proposed ACL model.

The initial learning rate, max epochs, validation frequency, and minimum batch size, which are training option parameters of the ACL model, are selected as 0.001, 5, 30 and 32, respectively. The training optimization solver was stochastic gradient descent with momentum (SGDM). More detailed information about the simulation parameters performed is given in Table A1 in Appendix A. The Matlab integrated development environment (IDE) containing the proposed approach coding was run for 70–30%, 80–20%, and 90–10% training-test ratios. Accuracy and loss graphs in training-test processes for these options are given in Figure 5.

As seen in Figure 5, training-test accuracy and training-test loss values are given for all training-test ratios. The training accuracies for all training-test ratios were 100%. The best test accuracy (100%) was obtained for the 90–10% training-test ratio, while the worst test accuracy (94.65%) was obtained for the 70–30% training-test ratio. The best training-test loss values (0.019–0.01) were obtained for the 90–10% training-test ratio, while the worst training-test loss values (0.12–0.16) were obtained for the 70–30% training-test ratio.

**Figure 6:**
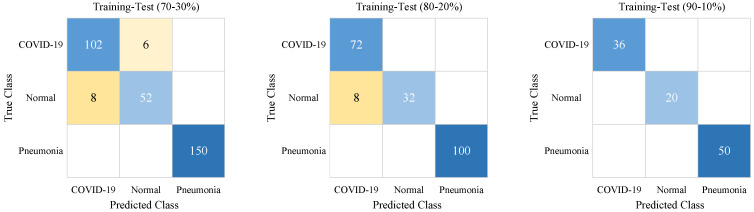
The confusion matrices for different training-test ratios of the proposed ACL model. The highest true and false values have a darker background.

At the end of the training process, according to class names, the test confusion matrix results are given in Figure 6 for different training-test ratios.

As seen in Figure 6, pneumonia samples were predicted with 100% accuracy. The worst COVID-19 and Normal sample predictions were obtained for the 70–30% training-test ratio. The COVID-19 samples were predicted with 100% accuracy for the 80–20% and 90–10% training-test ratios. The best prediction for Normal samples was achieved with the 90–10% training-test ratio.

In Table 2, the results of performance metrics, which consisted of sensitivity (*Se*), specificity (*Sp*), precision (*Pr*), and *F*-*score*, are given for different training-test ratios of the proposed ACL model. Using true positive (*TP*), true negative (*TN*), false positive (*FP*), and false negative (*FN*) values, these performance metrics were calculated in Equations (11)–(14) as follows:

(11) $$Se=\frac{TP}{TP+FN}$$

(12) $$Sp=\frac{TN}{TN+FP}$$

(13) $$Pr=\frac{TP}{TP+FP}$$

(14) $$F\text{-}score=\frac{2\times TP}{2\times TP+FP+FN}$$

In the 70–30% training-test ratio, the best Se was 1.0 for the Pneumonia class and the worst Se was 0.87 for the Normal class. The best Sp was 1.0 for the Pneumonia class and the worst Sp was 0.96 for the COVID-19 class. The best Pr was 1.0 for the Pneumonia class and the worst Pr was 0.90 for the Normal class. The best F-score was 1.0 for the Pneumonia class and the worst F-score was 0.88 for the Normal class. In the 80–20% training-test ratio, all metric results of the Pneumonia class were 1.0. The Se (1.0) of the COVID-19 class outperformed the Normal class. The worst Sp (0.94) and Pr (0.90) were obtained with the COVID-19 class while the worst F-score (0.89) was obtained with the Normal class. In the 90–10% training-test ratio, all metric results were 1.0 for all classes.

**Figure 7:**
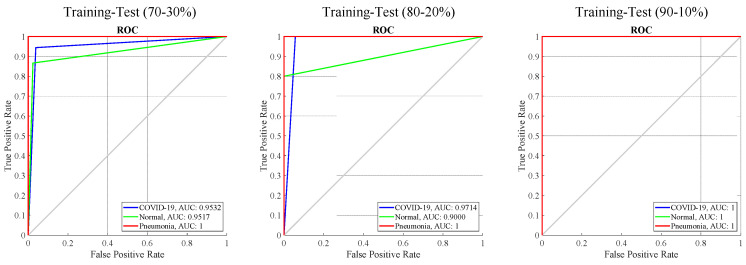
The ROC curves and AUC values for different training-test ratios of the proposed ACL model.

In Figure 7, ROC graphs and AUC results are given for all training-test ratios. The AUC values of the Pneumonia class were 1.0 in all training-test ratios. For the 70–30% training-test and 80–20% training-test, the COVID-19 class AUC results were 0.9532 and 0.9714, respectively. For the 70–30% training-test and 80–20% training-test, the Normal class AUC results were 0.9517 and 0.9000, respectively. In the 90–10% training-test ratio, the AUC values were 1.0 for the COVID-19 and Normal classes.

## 7. Discussion

**Figure 8:**
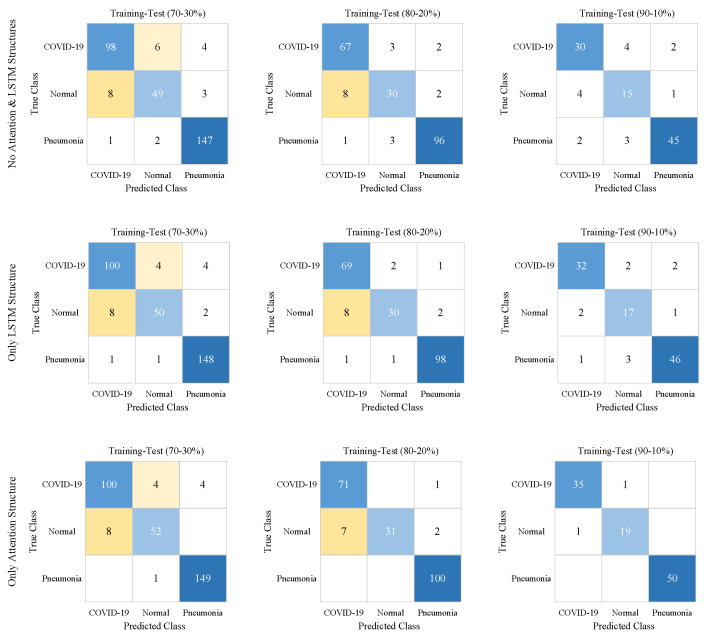
The confusion matrices show the effects of Attention and LSTM structures on the proposed CNN model. The highest true and false values have a darker background.

In Figure 8, for all training-test ratios, confusion matrix results are given to evaluate the performance of the attention strategy and LSTM structure. In Table 3, the performance metrics results are calculated using TP, TN, FP, and FN values in these confusion matrices.

As seen in Table 3, attention strategy and LSTM structure, which synchronously operated in the ACL model, improved all performance metrics for all training-test ratios. The worst performance metrics results were obtained with the CNN model (Case 1) without the attention strategy and LSTM structure. The CNN model (Case 3) with only the attention strategy outperformed the CNN model (Case 2) with the only LSTM structure. According to the models in cases 1, 2, and 3, in the 70–30% training-test ratio, the Acc scores of the ACL model (Case 4) were improved by 4%, 3%, and 2%, respectively. In the 80–20% training-test ratio, the Acc scores of the ACL model were improved by 5%, 3%, and 1%, respectively. In the 90–10% training-test ratio, the Acc scores of the ACL model were improved by 15%, 10%, and 2%, respectively. For 70–30%, 80–20%, and 90–10% training-test ratios, the classification accuracies of MCW images compared to raw images were improved by 2%, 4%, and 5%, respectively.

**Figure 9:**
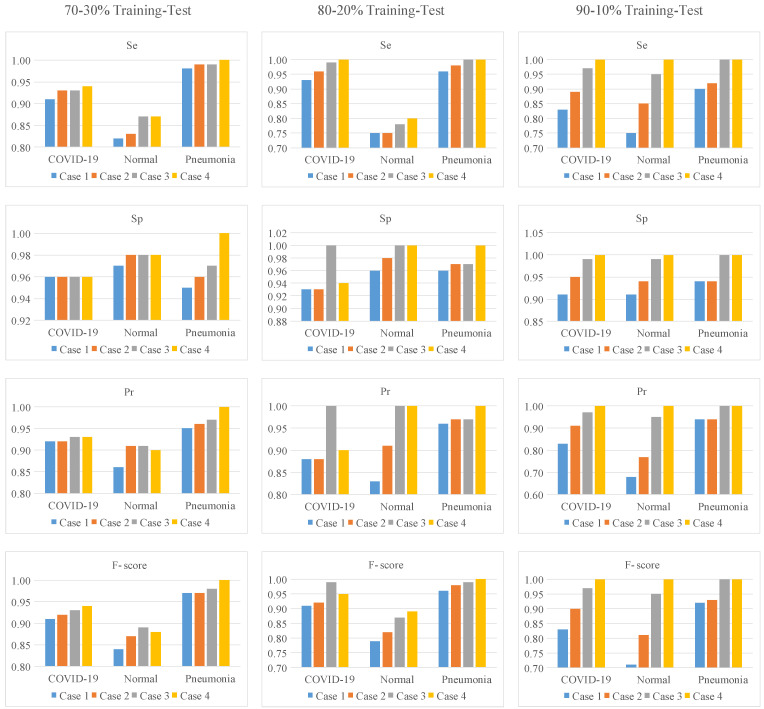
The graphical analysis of performance metrics given in Table 3 for all training-test ratios.

To interpret the performance metrics in Table 3 more clearly, the graph in Figure 9 was created from the values in Table 3.

**Figure A1:**
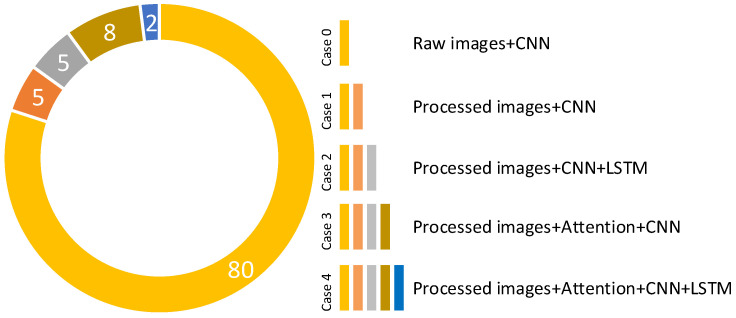
The contribution chart of the proposed methodology strategies.

As seen in Figure 9, the slope is positive for most performance metrics, given that a curve is fitted from Case 1 to Case 4. This means that the proposed approach improves the classification performance in these metric values. However, the slope from Case 1 to Case 4 was zero for the Sp metric in the COVID-19 class at a training-test rate of 70–30%. In other words, classification performance was not improved for this metric and class. The slope from Case 3 to Case 4 was negative for the Sp, Pr, and F-score metrics in the COVID-19 class at the 80–20% training-test ratio. The proposed approach achieved worse classification performance for the COVID-19 class on these metrics than the model in Case 3. Contributions of the MCW segmentation algorithm, attention structure, and LSTM model in the proposed approach are given in Figure A1 of Appendix A.

**Figure 10:**
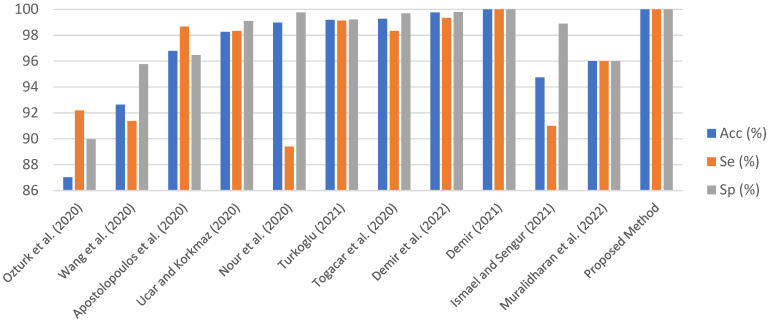
The bar graph obtained from Table 4 [9,19,21,24,25,26,27,41,42,43,44].

In Table 4, the proposed approach was compared to the state-of-the-art techniques. These existing studies are included in Table 4 for two reasons. First, these studies have been popular in the COVID-19 field. Second, other methods were added due to their high performance. Acc, Se, and Sp metrics in Table 4 were taken into consideration as they are common metrics in all studies. The bar graph in Figure 10 was created using the data in Table 4 to better examine the performance results among existing studies. It cannot be said that the proposed approach and the existing studies are completely superior to each other. This is because the COVID-19 dataset is not standardized, and training-test ratios and model training parameters are different.

Ozturk et al. [21] used a deep CNN model, which included the end-to-end learning strategy, for automated ILD classification. This model, named the DarkCovidNet, reached an accuracy of 87.02%. This study, which was first published in the scope of COVID-19, can be considered one of the baseline models. In Ref. [9], ILD was automatically detected from chest X-ray images using an end-to-end-trained CNN architecture with numerous residual blocks. ResNet-50 and VGG-19 CNN models were not as effective as this model. With this approach, the scores for Acc, Se, and Sp were 92.64%, 91.37%, and 95.76, respectively. In Ref [19], the Acc, Sp, and Se metrics were used to compare the performance of transfer learning models such MobileNet v2, VGG19, and Inception. The MobileNet v2 model produced the best results. For automated ILD diagnosis, a SqueezeNet Model trained with the enhanced dataset from scratch was suggested in Ref. [41]. Additionally, hyperparameter optimization employed the Bayesian approach. Values of 98.26%, 98.33%, and 99.10%, respectively, were the highest ones recorded for Acc, Se, and Sp. In Ref [42], deep features from chest X-ray images were extracted using an end-to-end-trained CNN model with five convolutional layers. The SVM classifier with radial basis function kernel achieved an Acc of 98.97%, an Se of 89.39%, and an Sp of 99.75 during the classification stage. Deep features from the fully connected and convolutional layers of the AlexNet model were retrieved for Ref [43]. The Relief algorithm decreased a total of 10,568 deep features to 1500 deep features. This model had 99.18% Acc, 99.13% Se, and 99.21% Sp, respectively. In Ref [44], MobileNet v2 and SqueezeNet models were used to create the integrated features. The SVM classifier achieved an Acc of 99.27%, Se of 98.33%, and Sp of 99.69% after hyperparameters were tweaked with the Social Mimic method. Seven convolutional layers of the compressed CA model were used in the DeepCov19Net Model [27] to extract deep features. Three techniques were utilized in the pre-processing (Laplacian), feature selection (SDAR), and hyperparameter tuning (Bayesian) stages to improve classification performance. With an accuracy of 99.75%, sensitivity of 99.33%, and specificity of 99.79%, the suggested approach performed well. Convolutional layers and the LSTM model were merged in Demir’s hybrid deep learning model [24] to automatically detect ILD. The DeepCoroNet model achieved a classification accuracy of 96.54%. For ILD classification, Ismael and Sengur [25] employed a ResNet50 based-transfer learning method. The ResNet50 model’s deep characteristics were taken. The SVM method was able to classify ILD samples with deep features with an overall accuracy of 94.7%. An innovative deep learning technique was used by Muralidharan et al. [26] to detect ILD automatically from X-ray images. First, the fixed boundary-based two-dimensional empirical wavelet transform (FB2DEWT) approach was used to fine-tune X-ray image levels with seven modes. These multiscale images were sent to the multiscale deep CNN to classify healthy, COVID-19, and pneumonia samples. Using this model, an accuracy of 96% was achieved.

The accuracy (100%) of the proposed approach is valid for a 90–10% training-test ratio. As this ratio is decreased, it has been observed that the classification performance decreases. In addition, the limitation of sample input sizes to 100 × 100 also affected the classification performance. Classification performance can be improved by increasing the input size with more powerful hardware.

The datasets used in this study were brought together from three different sources. This limits a more realistic performance comparison with existing studies. Evaluations that will be made with samples obtained from a more organized and single database will be able to make more reliable performance comparisons.

## 8. Conclusions

In this study, ILD classification was performed with a powerful customized deep learning-based method. In the proposed approach, the MCW segmentation algorithm, which emphasizes the spots and traces in CX-R images in the COVID-19 class, is used for a more efficient operation of the attention structure in the ACL model. Attention and LSTM architectures in the ACL model have increased the classification performance as mentioned in the Discussion section. The classification performance of the model was evaluated for different training-test ratios. Classification accuracy reached 100% at a test rate of 90–10%. At test rates of 80–20% and 70–30%, the success rate was over 96%. The performance of the model was compared with both baseline and high classification methods. Although the classification performance of the proposed approach is good according to these methods, it is not correct to talk about the superiority of the methods because the data sets and evaluation methods used are not the same. The classification performance was obtained with low-size input data such as 100 × 100. If the hardware performance is further increased, it is possible to increase the classification performance even more. Additionally, it has been seen that the hyperparameter selection in the proposed deep learning model is very important in classification performance. These hyperparameters are tuned for empirical outputs. In future studies, the hyperparameters of deep learning models will be automatically tuned by optimization techniques such as the Bayesian optimization algorithm.

## Figures and Tables

**Table 1:** The architecture information of the proposed ACL model.

Layer #	Layer Name	Layer	Layer Info
1	input	Sequence Input	Sequence input with 100 × 100 × 3 dimensions
2	fold	Sequence Folding	Sequence folding
3	convInp2d_1	Convolution	16 3 × 3 × 3 convolutions with stride: 1 and padding: 0
4	batchnorm_1	BN	BN with 16 channels
5	relu1	ReLU	ReLU
6	maxpool2d_1	Max Pooling	3 × 3 max pooling with stride: 1 and padding: 0
7	convInp2d_2	Convolution	16 3 × 3 × 16 convolutions with stride: 1 and padding: 0
8	batchnorm_2	BN	BN with 16 channels
9	relu2	ReLU	ReLU
10	maxpool2d2	Max Pooling	3 × 3 max pooling with stride: 1 and padding: 0
11	convInp2d_3	Convolution	16 3 × 3 × 16 convolutions with stride: 1 and padding: 0
12	relu_2_1_1_5	ReLU	ReLU
13	convInp2d_4	Convolution	16 3 × 3 × 16 convolutions with stride: 1 and padding: 0
14	maxpool2d_2	Max Pooling	3 × 3 max pooling with stride: 1 and padding: 0
15	convInp2d_5	Convolution	16 3 × 3 × 16 convolutions with stride: 1 and padding: 0
16	relu_2_1_1_6	ReLU	ReLU
17	convInp2d_6	Convolution	16 3 × 3 × 16 convolutions with stride: 1 and padding: 0
18	sigmoid_1_1_1_3	sigmoidLayer	sigmoidLayer
19	mul_1_1_1_3	ElementWiseMultiplication	Element Wise Multiplication of 2 inputs
20	unfold	Sequence Unfolding	Sequence unfolding
21	flatten	Flatten	Flatten
22	lstm	LSTM	LSTM with 100 hidden units
23	fc0	FC	100 FC layer
24	ReLu2	ReLU	ReLU
25	drop1	Dropout	40% dropout
26	fc1	FC	3 FC layer
27	softmax	Softmax	softmax
28	class output	Classification Output	crossentropyex

**Table 2:** The performance metrics for different training-test ratios of the proposed ACL model.

Training-Test Ratios	Classes	Se	Sp	Pr	F-score
70–30%	COVID-19	0.94	0.96	0.93	0.94
	Normal	0.87	0.98	0.90	0.88
	Pneumonia	1.0	1.0	1.0	1.0
80–20%	COVID-19	1.0	0.94	0.90	0.95
	Normal	0.80	1.0	1.0	0.89
	Pneumonia	1.0	1.0	1.0	1.0
90–10%	COVID-19	1.0	1.0	1.0	1.0
	Normal	1.0	1.0	1.0	1.0
	Pneumonia	1.0	1.0	1.0	1.0

**Table 3:** The performance scores that show the effects of Attention and LSTM structures on the proposed CNN model.

Case #	Models	Training-Test Ratios	Classes	Se	Sp	Pr	F-score	Acc
1	No Attention and LSTM Structures	70–30%	COVID-19	0.91	0.96	0.92	0.91	0.92
			Normal	0.82	0.97	0.86	0.84	
			Pneumonia	0.98	0.95	0.95	0.97	
		80–20%	COVID-19	0.93	0.93	0.88	0.91	0.91
			Normal	0.75	0.96	0.83	0.79	
			Pneumonia	0.96	0.96	0.96	0.96	
		90–10%	COVID-19	0.83	0.91	0.83	0.83	0.85
			Normal	0.75	0.91	0.68	0.71	
			Pneumonia	0.9	0.94	0.94	0.92	
2	Only LSTM Structure	70–30%	COVID-19	0.93	0.96	0.92	0.92	0.94
			Normal	0.83	0.98	0.91	0.87	
			Pneumonia	0.99	0.96	0.96	0.97	
		80–20%	COVID-19	0.96	0.93	0.88	0.92	0.93
			Normal	0.75	0.98	0.91	0.82	
			Pneumonia	0.98	0.97	0.97	0.98	
		90–10%	COVID-19	0.89	0.95	0.91	0.90	0.90
			Normal	0.85	0.94	0.77	0.81	
			Pneumonia	0.92	0.94	0.94	0.93	
3	Only Attention Structure	70–30%	COVID-19	0.93	0.96	0.93	0.93	0.95
			Normal	0.87	0.98	0.91	0.89	
			Pneumonia	0.99	0.97	0.97	0.98	
		80–20%	COVID-19	0.99	1.0	1.0	0.99	0.95
			Normal	0.78	1.0	1.0	0.87	
			Pneumonia	1.0	0.97	0.97	0.99	
		90–10%	COVID-19	0.97	0.99	0.97	0.97	0.98
			Normal	0.95	0.99	0.95	0.95	
			Pneumonia	1.0	1.0	1.0	1.0	
4	Proposed Approach	70–30%	COVID-19	0.94	0.96	0.93	0.94	0.96
			Normal	0.87	0.98	0.90	0.88	
			Pneumonia	1.0	1.0	1.0	1.0	
		80–20%	COVID-19	1.0	0.94	0.90	0.95	0.96
			Normal	0.80	1.0	1.0	0.89	
			Pneumonia	1.0	1.0	1.0	1.0	
		90–10%	COVID-19	1.0	1.0	1.0	1.0	1.0
			Normal	1.0	1.0	1.0	1.0	
			Pneumonia	1.0	1.0	1.0	1.0	

**Table 4:** The comparison of the proposed approach compared to existing methodologies.

Authors	Methods	Dataset	Classes #	Acc (%)	Se (%)	Sp (%)
Ozturk et al. [21]	DarkCovidNet	Public	3	87.02	92.18	89.96
Wang et al. [9]	COVID-Net	Public	3	92.64	91.37	95.76
Apostolopoulos et al. [19]	The pre-trained CNNs	Public	3	96.78	98.66	96.46
Ucar and Korkmaz [41]	COVIDiagnosis-Net	Public	3	98.26	98.33	99.10
Nour et al. [42]	Deep CNN, SVM	Public	3	98.97	89.39	99.75
Turkoglu [43]	AlexNet, Feature Selection, SVM	Public	3	99.18	99.13	99.21
Togacar et al. [44]	Deep features, SqueezeNet, SVM	Public	3	99.27	98.33	99.69
Demir et al. [27]	DeepCov19Net	Public	3	99.75	99.33	99.79
Demir [24]	DeepCoroNet	Public	3	100.00	100.00	100.00
Ismael and Sengur [25]	ResNet50 Features + SVM	Public	2	94.74	91.00	98.89
Muralidharan et al. [26]	FB2DEWT + CNN	Public	3	96.00	96.00	96.00
Proposed Method	Processed images, ACL model	Public	3	100.00	100.00	100.00

## Data Availability

In this paper, the dataset is publicly available.

## Appendix A

**Table A1:** Training option parameters of the SGDM solver.

SGDM Options	Values
Momentum	0.9
Initial Learn Rate	0.001
Learn Rate Schedule	‘none’
Learn Rate Drop Factor	0.1
Learn Rate Drop Period	10
L2 Regularization	0.0001
Gradient Threshold Method	‘l2norm’
Gradient Threshold	Inf
Max Epochs	5
Mini-BatchSize	32
Verbose	0
Verbose Frequency	50
Validation Frequency	30
Validation Patience	Inf
Shuffle	‘every-epoch’
Execution Environment	‘auto’
Sequence Length	‘longest’
Sequence Padding Value	0
Sequence Padding Direction	‘right’
Dispatch In Background	0
Reset Input Normalization	1
Batch Normalization Statistics	‘population’
